# The Law Regulating Dental Practice

**Published:** 1868-08

**Authors:** 


### Editorial.

### THE LAW REGULATING DENTAL PRACTICE.

So many enquiries continue to be made in reference to the force and bearing of the law pertaining to the practice of Dentistry, that we embrace this as the easiest method of replying. We trust that the profession will readily apprehend, that the Board of Examiners under this law have a specified duty to perform, viz: to examine and decide upon the qualifications of those who may apply, or those who cannot legally practice without the certificate of the Board; and here the official duty of this Board ends.

But, the members of the Board may, if they choose, give their opinion upon various points of enquiry that may arise, and that opinion will be just as good as that of any other person equally informed, and no better. Nevertheless, we are always willing to assist any out of difficulty when we can. And as we have been somewhat involved in legal matters for the profession for a year or two past, we have occasionally referred some of these vexed questions to those upon whose legal knowledge we have been accustomed to rely.

Dr. P, enquires: "Can one without a Diploma, who practiced four years previous to January, 1867, and at that time ceased to practice, and engaged in other business, now resume the practice of Dentistry in the State of Ohio, without violating the law for the regulation of the practice of Dentistry in this State, without a certificate from the Board of Examiners?"

The law makes no such exceptions, it says:

SECTION 1. *Be it enacted by the General Assembly of the State of Ohio*, That it shall be unlawful for any person to practice Dentistry in the State of Ohio for compensation, unless such person has received a Diploma from the faculty of a Dental College, duly incorporated under the laws of this or any other State of the United States, or foreign country, or a certificate of qualification issued by the State Dental Society, or by any local Society auxiliary thereto; provided that nothing in this section shall apply to persons *now* engaged in the practice of Dentistry in this State, before the first day of January, 1873. The law was passed on the 6th of May, 1868.

We regard this as a decisive answer to the enquiry.

Another asks: "Can one who commenced five years ago, with a regular practice, and has confined himself to extracting teeth, taking impressions, and fitting in artificial teeth exclusively ever since—possibly has attempted to fill a dozen teeth in the time—be regarded in the eyes of the law as a practicing Dentist; and would he violate the law by opening an office for general Dental practice, without having obtained a diploma, or a certificate of the State Board?"

We doubt not it would be a clear violation of both the spirit and letter of the law, and would be so held by any court. The fact of such an one having been in business connection, with a regular practitioner, would in no way change the aspect of the case; for admitting that, then one totally ignorant of the practice of Dentistry could attach himself by a pecuniary partnership to a regular practitioner (which is sometimes done, and properly too) and afterward withdraw, and then claim to practice upon his own responsibility in accordance with the law. The clear intent and spirit of the law is, that any one practicing under its restrictions, shall be competent.

The law clearly defines the class of persons who do not come under its restrictions, viz: "Provided that nothing in this section shall apply to persons *now* engaged in the practice of Dentistry in this State, before the first day of January, 1873." Now, does any one for a moment suppose that the extraction of teeth alone, or in addition, taking impressions and fitting artificial teeth, would be regarded by any court as practicing Dentistry according to the intent of the law? Most certainly not.

Another says: "I have been in practice in an adjoining State for several years. I have made arrangements to go into practice immediately in Ohio, and I have no diploma, and the next meeting of the Board is not until December next. What shall I do?"

In such case we would suggest, that if six or more applicants should request it, a meeting of the Board could be called at any time within a few days, and at least a quorum be got together.

This would meet cases of this kind, and we will here suggest to any who desire to appear before the Board at the earliest possible time, that they notify some member of the Board, and so soon as the number above indicated shall make such application, a meeting will be called.

It is very probable that any operator of good ability and attainments, being able to present the evidence of good professional standing in his former locality, and manifesting a proper professional bearing and deportment, might enter upon practice without interruption, with the full understanding that he would go before the Board at the first opportunity. Of course he would incur the risk of prosecution, which, under favorable circumstances, would perhaps be but slight. In such cases as the above, we have no advice to give, but only speak suggestively.

Again, it is asked: "Do those operating—without a diploma or license—as employees of a qualified Dentist, violate the law?" Most certainly not. Employers are always responsible for the acts of employees.

We presume as little inconvenience will be caused by this law as by any one that would affect so large a number of persons; but the benefit that will ultimate to community, will a thousand-fold exceed all such inconvenience. T.

